# Comparison of Photochemically Sealed Commercial Biomembranes for Nerve Regeneration

**DOI:** 10.3390/jfb16020050

**Published:** 2025-02-06

**Authors:** Maria Bejar-Chapa, Nicolò Rossi, Nicholas C. King, David M. Kostyra, Madison R. Hussey, Kalyn R. McGuire, Mark A. Randolph, Robert W. Redmond, Jonathan M. Winograd

**Affiliations:** 1 Division of Plastic and Reconstructive Surgery, Department of Surgery, Massachusetts General Hospital, Harvard Medical School, Boston, MA 02114, USA; 2 Wellman Center for Photomedicine, Massachusetts General Hospital, Boston, MA 02114, USA

**Keywords:** nerve regeneration, amniotic membrane, sciatic nerve, photochemical tissue bonding, microsurgery

## Abstract

Peripheral nerve injuries affect 13–23 per 100,000 people annually in the U.S. and often result in motor and sensory deficits. Microsurgical suture repair (SR) is the standard treatment but is technically challenging and associated with complications. Photochemical tissue bonding (PTB), which uses light and a photoactivated dye to bond collagenous tissues, offers a promising alternative. We compared PTB with commercially available collagen membranes for SR and PTB using cryopreserved human amnion (HAM) in a rat sciatic nerve transection model. In total, 75 Lewis rats underwent nerve repair with one of five methods: SR, PTB-HAM, PTB with commercial collagenous membranes (human amnion monolayer (AML), human amnion–chorion–amnion trilayer (ATL), or swine intestinal submucosa (SIS)). Functional recovery was assessed with walking tracks and the Static Sciatic Index (SSI) at days 30, 60, 90, and 120; histological evaluations at days 30 and 120 examined inflammation, axon density, and fascicle structure. No significant differences in SSI scores were found between groups, though PTB-AML and PTB-SIS improved over time. Histology showed inflammation at day 30 that decreased by day 120. Histomorphometry revealed similar axon regeneration across groups. These results suggest that PTB with commercial membranes is a viable alternative to SR.

## 1. Introduction

Peripheral nerve injuries often lead to significant impairment in function and quality of life, including prolonged motor and sensory deficits, as well as the formation of painful neuromas. In the United States, an estimated 13 to 23 cases of nerve injury per 100,000 people occur each year [1]. Furthermore, several studies have reported an increase in peripheral nerve injuries (PNI), with their incidence rising from 1.64% in 1998 to 2.3% in 2015 [2,3]. The current gold standard for treating peripheral nerve transection injuries is microsurgical suture repair (SR). Its advantages include precise coaptation and a low risk of dehiscence [4,5,6]. However, SR is technically demanding, time-consuming, and is frequently associated with postoperative inflammation, intra- and extra-neural scarring, and axon loss to the periphery of the repair site, all of which hinder nerve regeneration [7,8,9,10]. Consequently, alternative nerve repair strategies have been investigated. One method is fibrin glue, first introduced as a treatment for nerve injuries in 1940 [11,12], with some studies showing better functional outcomes than suture in animal models [13], but a similar recovery rate to that of suture in humans [14]. Even though it reduces the operative time and the formation of scar tissue, the bond strength of fibrin glue is poor and its rate of dehiscence high (6–80%), and, therefore, it has remained an off-label surgical treatment for nerve injury [15].

Bioengineered conduits (e.g., bovine collagen type I, polyglycolic acid (PGA), polycaprolactone (PCL), polyvinyl alcohol (PVA), and porcine small intestine submucosa) have been used to create or reinforce nerve repairs with no gaps and gaps <5 mm [16]. Although conduit repair is performed using sutures to remove tension (whereby the proximal and distal nerve ends are secured within the conduit), it provides no immediate seal to the regenerative environment within the repair site. It has also been associated with a higher frequency of adverse events and the need for revision surgery when compared to standard suture neurorrhaphy, and the evidence to recommend conduits over standard sutures in humans is uncertain [17].

One promising method for nerve repair is photochemical tissue bonding (PTB), in which green light and a photoactivated dye are used to bond collagenous tissues via crosslinking. Its efficacy has been demonstrated for repair of peripheral nerves, as well as skin, bowel, blood vessels, and dura mater [18,19,20,21,22,23,24,25,26,27,28]. The current technique consists of application of aqueous Rose Bengal, a photosensitizing fluoresceine dye currently approved by the FDA and used topically for corneal staining, to the tissue surface, followed by irradiation with 532 nm light. This promotes local oxidation of exposed tissue collagen, which results in cross-linking that generates a bio-mimetic bond [29]. As applied to peripheral nerve repair, PTB has most commonly been used to bond a preserved human amniotic membrane wrap around the transected apposed nerve ends. This creates a watertight seal that is theorized to better contain neuro-regenerative growth factors, prevent axonal escape, and minimize adhesions at the repair site [18,20].

To date, there are no known systemic side effects that can be attributed to the use of PTB and HAM for end-to-end neurorrhaphy as performed in this study. Local side effects that would manifest as altered nerve regeneration or intraneural scarring above the amount typically seen with standard repair are analyzed in this study. Its safety has been established in multiple prior nerve photosealing studies in rats, rabbits, and non-human primates (NHP), where either equivalent or enhanced regeneration have been shown in comparison to standard repair [18,19,20,26,27,30,31,32].

During nerve repair, adhesions are formed by intra- and extra-neural scar tissue originating from infiltration of fibroblasts and myofibroblasts [33,34]. Intraneural scarring can lead to impairments in nerve regeneration, while external scarring creates adhesions, which can cause impaired nerve function, including pain, sensory changes, and potential weakness in the distribution of the affected nerve [35]. Currently, neurolysis is indicated to remove pathologic scar tissue around the nerve caused by trauma or surgery. Strategies such as the application of vein wraps, adipofascial flaps, and commercially available membrane wraps (SIS, type 1 bovine collagen, HAM) are used to decrease nerve scar formation after revision surgery [36,37,38,39]. Similarly, previous studies have reported that PTB repair with human amnion decreases adhesion formation when compared to suture repair [18,19,20]. HAM is theorized to promote wound healing and improve nerve regeneration through several mechanisms [40,41], including: anti-fibrosis via inhibition of the TGF-b signal responsible for fibroblast activation and differentiation [42,43]; anti-inflammation via the presence of interleukins IL-4, 6, 8, and 10, inhibition of neutrophil and macrophage chemotaxis, and suppression of B and T lymphocyte proliferation [40,44]; and low antigenicity due to lack of human leukocyte antigens HLA-A, B, C, and DR on its epithelial cell surface [45].

Studies utilizing PTB with human amniotic membrane (PTB/HAM) have consistently shown superior histological and functional outcomes compared to suture repair across various animal models. In a rodent sciatic nerve transection injury model followed for 12 weeks, PTB demonstrated significant advantages, including larger fiber size and thicker myelination of regenerating fibers and improved functional recovery [18]. PTB is compatible with both autografts and allografts for repairing longer nerve gaps, though in those cases, the HAM is crosslinked (xHAM) to slow the process of enzymatic degradation to allow more time for nerve regeneration and healing at the neurorrhaphy site [18,19,20,21]. For example, in a model of a 15 mm nerve gap injury, combining autografts with PTB/xHAM yielded superior results compared to autografts repaired with sutures alone at a 5-month follow-up [19]. Similar success was observed in models of immediate or delayed nerve injuries repaired with autografts and PTB/xHAM versus suture repair at the same follow-up interval [20]. Furthermore, allografts paired with PTB/xHAM also demonstrated favorable outcomes in a separate large nerve gap injury model, assessed at a 5-month follow-up, with functional recovery approaching that of sutured autografts [26].

While PTB has traditionally been paired with amnion harvested from human placenta and cryopreserved before use, a recent study found that PTB repairs using commercially available human amnion products and other collagenous membranes exhibit biomechanical properties comparable to those of cryopreserved human amnion [22]. The advantages of commercial membranes include clinical availability, FDA approval, and no risk of bloodborne pathogen transmission. However, their efficacy in nerve regeneration models is not yet established. The aim of this study was to compare the axonal regeneration and functional outcomes of PTB nerve repairs using HAM, known for its efficacy, with repairs using commercially available collagenous membranes in a standard rodent model.

## 2. Materials and Methods

### 2.1. Animals

This protocol was reviewed and approved by the Massachusetts General Hospital Institutional Animal Care and Use Committee (IACUC) and the Animal Care and Use in Research Office (ACURO) of the US Army Medical Research and Development Command. All animals were housed, cared for, and used in compliance with the Guide for the Care and Use of Laboratory Animals (National Research Council, 2011) [46], in a program accredited by the Association for the Assessment and Accreditation of Laboratory Animal Care, International. The use of human amniotic membrane (HAM) from human placenta was approved by the Massachusetts General Hospital Institutional Review Board. Integra LifeSciences provided material at no cost, and additional material was purchased by Massachusetts General Hospital from Smith and Nephew to conduct this study.

A total of 75 male Lewis rats (Charles River Laboratories) were randomized into one of five experimental groups, each with *n* = 15 rats. The average age and weight of the animals was 11.3 ± 1.4 weeks and 300.3 ± 27.2 g, respectively. The weight selection for our study was based on previous studies and maintained consistency with established protocols at our laboratory. This weight range is optimal for the given rat size, as it minimizes the challenges associated with neurorrhaphy, ensuring more reliable surgical outcomes. It has been found that the greater the weight of the rats, the higher the challenge for nerve regeneration [47].

The rats were housed in pairs and acclimated for at least 24 h prior to surgery under standard laboratory conditions (20.5 °C, humidity 30–70%, 12:12 h light–dark cycle). Food and water were provided ad libitum. For each experimental group, the right sciatic nerve was transected and then repaired using one of the following methods: (1) SR with six 10-0 nylon epineurial sutures, (2) PTB with non-crosslinked cryopreserved human amnion [18], or PTB with one of the following commercial membranes: (3) human amnion monolayer (AML) (AmnioExcel^®^, Integra LifeSciences, Princeton, NJ, USA (4) human amnion-chorion-amnion trilayer (ATL) (AmnioExcel^®^ Plus, Integra Life Sciences Princeton, NJ, USA), or (5) swine intestinal submucosa (SIS) (Oasis^TM^, Smith and Nephew, London, UK). These commercial membranes were selected for their high collagen content and their origin from cryopreserved amnion (except for SIS). They were also chosen for their dehydrated, acellular nature, which enables room-temperature storage and eliminates the risk of blood-borne pathogen infection. Furthermore, these membranes have demonstrated favorable biomechanical properties in prior in vitro testing [22].

Five rats per group were euthanized at post-operative day 30 (POD30) for histological analysis, and ten rats per group were euthanized at POD120 for histological analysis and histomorphometry.

### 2.2. Surgical Procedure

Under isoflurane anesthesia, the sciatic nerve was dissected and transected 8 mm proximal to the nerve trifurcation. For all PTB repair groups, two 10-0 nylon epineurial sutures were placed at the neurorrhaphy. The experimental membrane was cut into a rectangle of 8 × 6 mm, placed in PBS for 10 min, and dried with a cotton swab. Rose Bengal dye (Aldrich, Milwaukee, WI, USA), 0.1% *w*/*v* in sterile PBS, was applied to the experimental membranes for absorption. The membrane was covered to protect it from light for 4 min to avoid photodegradation of the dye itself. The membrane was then wrapped around the neurorrhaphy with approximately 50% overlap, with the long dimension of the amnion oriented longitudinally and the RB-stained surface in contact with the epineurium. The repair site was exposed to green laser light at 532 nm from a continuous-wave KTP laser (Laserscope Aura-i, San Jose, CA, USA; irradiance of 0.4 W/cm^2^) for 2 min, followed by 180° rotation and repeat irradiation on the opposite side. For the SR group, following sciatic transection, six 10-0 nylon interrupted epineurial sutures were placed to create a standard neurorrhaphy. Post-operative monitoring occurred every 12 h for the first 72 h, and then once a week until the end of the study. Pain was managed with 0.05 mg/kg buprenorphine SC q/24 h for the first 72 h after surgery.

### 2.3. Outcome Assessment

#### 2.3.1. Functional Assessment—Walking Track Analysis

Walking tracks (WT) [48,49] were performed at baseline, and at postoperative days 30, 60, 90, and 120. These time points were selected to monitor changes in functional outcomes over time. Measuring at monthly intervals over a 4-month period allows the capture of meaningful changes at each stage, whereas more frequent measurements, such as weekly, may not provide additional insights and could be unnecessarily redundant. A 120-day follow-up is a standard observation period for direct nerve repair injury models in rats, as shorter time frames often fail to show significant improvements in functional outcomes. Extending the follow-up beyond this period typically provides limited additional insight. Moreover, this duration allows ample time for axons to cross the repair site, which typically occurs within 3 to 4 weeks [50].

To capture the rats’ footprints, their hind paws were immersed in ink, and they walked across a track where their prints were recorded on paper. The Static Sciatic Index (SSI) [51], a simplified version of the Sciatic Functional Index (SFI) that excludes print length from its calculation, was evaluated at each time point by two independent, blinded graders. The main reason for using the SSI formula instead of the SFI was because variations in footprint length have been shown to lead to misinterpretation and measurement errors.

In the Static Functional Index, the Toe Spread Factor (TSF), which is the measurement from toes 1 to 5, is the most significant (*p* < 0.0001), followed by the intermediary toe spread (ITS) factor (toes 2 to 4), which has near marginal significance (*p* = 0.0066), and it excludes the print length (PL) factor, due to its high variability and statistical insignificance (*p* = 0.3362). Similarly to the SFI, an index score of 0 was regarded as normal, while an index score of −100 represented complete impairment [51]. Rats sacrificed at POD30 (*n* = 5 per group) were excluded from the longitudinal SSI analysis.

#### 2.3.2. Histology and Histomorphometry

Histological examination was performed at postoperative days 30 and 120. Five rats from each of the five groups were euthanized at POD30. En bloc tissue samples of the sciatic nerve and muscle were sent for histological processing and staining with H&E to assess immune reaction to the different wraps. The remaining ten rats from each of the five groups were euthanized at 120 days. Five of these rats from each of the latter groups had their nerves and muscles harvested en bloc and stained with H&E to evaluate for immune reaction, and the other five rats had their experimental sciatic nerves harvested and processed for histomorphometry with toluidine blue (TB).

#### 2.3.3. Nerve Grading

**Figure 1:**
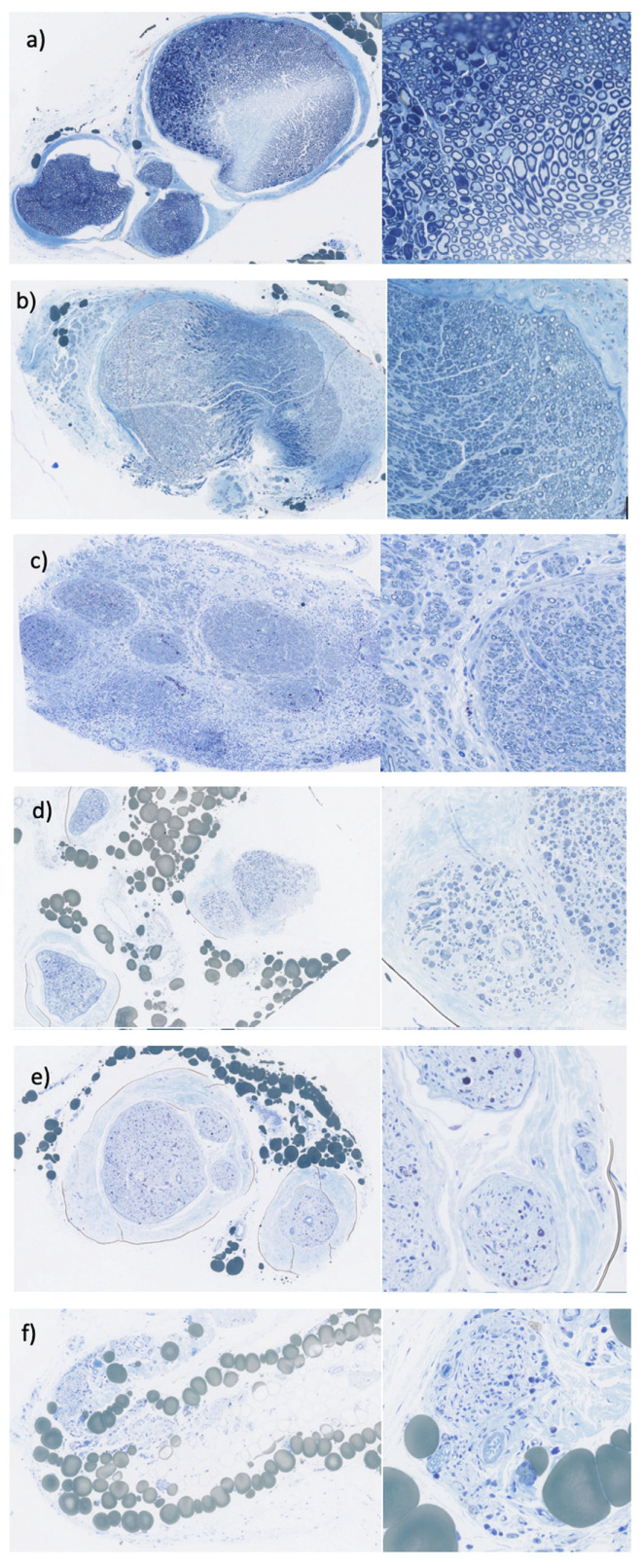
Nerve Semiquantitative Scoring System (NSQSS) measures the axon density within each nerve sample: (**a**) unoperated (control) nerve; (**b**) score 5: high density of axons similar to unoperated nerve; (**c**) score 4: approximately 75% axon density of a control nerve; (**d**) score 3: axon density approximately 50% of an unoperated nerve; (**e**) score 2: axon density is 25% of a control nerve; (**f**) score 1: very few or no axons present. Majority of tissue is connective tissue; 5x and 20x magnification are shown in each panel.

Our laboratory developed a nerve scoring system where comparisons of the surgically treated nerves are made to the contralateral unoperated nerve, based on a semi-quantitative assessment by three different blinded graders (Figure 1). This scoring system was found to be an efficient method of estimating the axon density of sampled nerves that is able to detect changes in axon composition over time [52].

The Nerve Semiquantitative Scoring System (NSQSS) evaluates the fascicle structure by assigning a binary score of 0 or 1 based on whether 2/3 or more of the axons are contained in well-defined fascicles. If 2/3 or more of the axons are contained in fascicles surrounded by perineurium, the sample receives a score of 1. If less than 2/3 of the axons are contained in fascicles the sample receives a score of 0. Axon density of the nerves is then graded on a 5-point scale reflecting overall axon count and density, with scores of 1–5 corresponding to 0%, 25%, 50%, 75%, and 100% of the axon density and count of a control (unoperated) nerve, respectively (Figure 1). This grading system accounts for average axon count and density; therefore, heterogeneity of axon distribution is not directly reflected in the scoring system. The scorers used their discretion to determine which score best matched the reference images in the grading system.

### 2.4. Statistical Analysis

All repair group SSI scores followed a normal distribution, as determined by the Shapiro–Wilks test with *p* > 0.01, with the exception of the AML repair group at post-op day 90 (*p* = 0.003), which, upon examination, was due to one rat with an SSI value trending towards baseline, likely representing near full recovery of function. The decision was made to assume a normal distribution for group SSI scores.

A mixed-effects model was run with SSI as the dependent variable, and the initial set of fixed predictors as: linear months in study (1–4, with baseline excluded), repair group (a categorical variable indexing the 5 repair methods used), and the 2-way interaction of those terms. The initial set of random effects were rat (nested in repair group), and the interaction between rat and month (permitted to be correlated). Nonsignificant higher-order terms were removed from the model to allow for more power to detect effects of interest. Residuals from fixed and random predicted values were checked for conformance to the model assumption of normality. Analysis of between-group differences was performed using ANOVA, with significance level *p* < 0.05. Analysis of within-group differences at different time points was performed using paired t-tests, with significance level *p* < 0.05.

The axon histology scores did not follow a normal distribution, and, therefore, analyses of differences between repair groups at proximal or distal sites were performed using the Kruskal–Wallis test, with significance *p* < 0.05. To evaluate whether distal axon scores were significantly lower than proximal scores within each repair group, a one-sided paired Wilcoxon test was implemented using the wilcoxon.exact package in RStudio (v. 2021.09.1), with significance *p* < 0.05 [53].

## 3. Results

### 3.1. Functional Recovery

The mixed-effects model of SSI scores showed a significant effect on SSI from month of +3.23 ± 0.70 (df = 1, F = 21.49, *p* < 0.001), but not from the repair group (df = 4, F = 0.70, *p* = 0.60). The interactions of group and month (fixed effect), and rat with month (random effect) were removed from the initial model due to nonsignificance.

**Figure 2:**
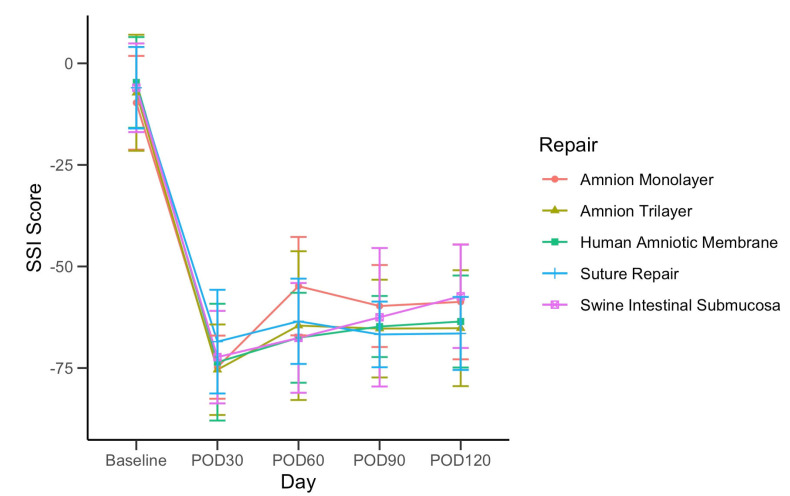
Average Static Sciatic Index (SSI), by repair group, recorded monthly from pre-op baseline to POD120. The error bars show standard deviations.

There was no significant difference between repair group SSI scores at any time point, as determined by ANOVA (*p* > 0.05) (Figure 2).

AML with PTB was the only group that showed a statistically significant increase in SSI scores from day 30 to day 60 (*p* = 0.002 for paired *t*-test), and this significant increase over day 30 persisted through days 90 and 120 (*p* < 0.05 for paired *t*-tests). The only other repair group with a significant increase in SSI scores from day 30 to 120 was SIS with PTB (*p* = 0.02).

### 3.2. Histology and Histomorphometry

**Figure 3:**
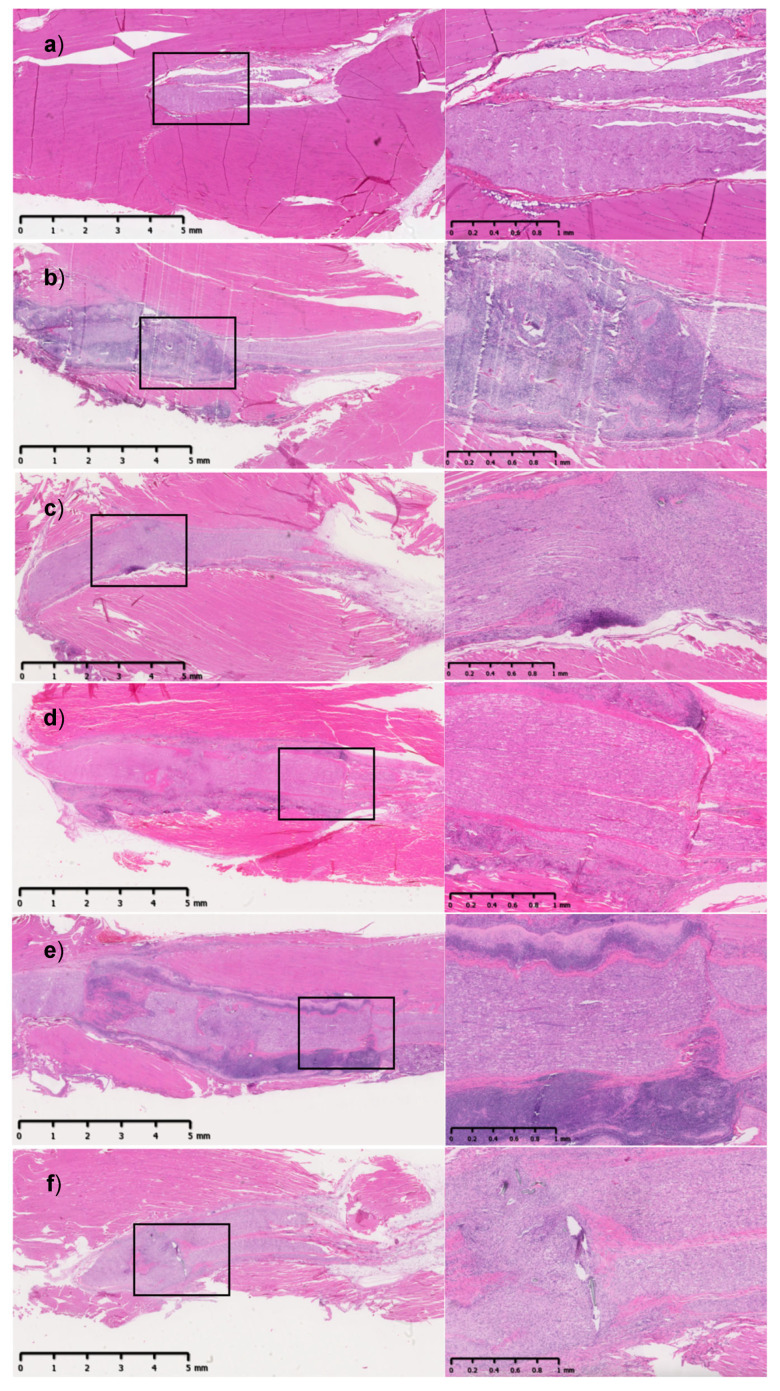
H&E-stained longitudinal sections of sciatic nerve and muscle (left 0.5x with scale bar to 5 mm, right 2x with scale bar to 1 mm) harvested at POD30. (**a**) UN: unoperated nerve, (**b**) AML: amnion monolayer, (**c**) ATL: amnion trilayer, (**d**) SIS: swine intestinal submucosa, (**e**) HAM: human amniotic membrane, (**f**) SR: suture repair. Note: the scale bar at the bottom shows 5 mm on the left, and 1 mm on the right column. The black boxes represent the enlarged image areas that are shown on the right.

**Figure 4:**
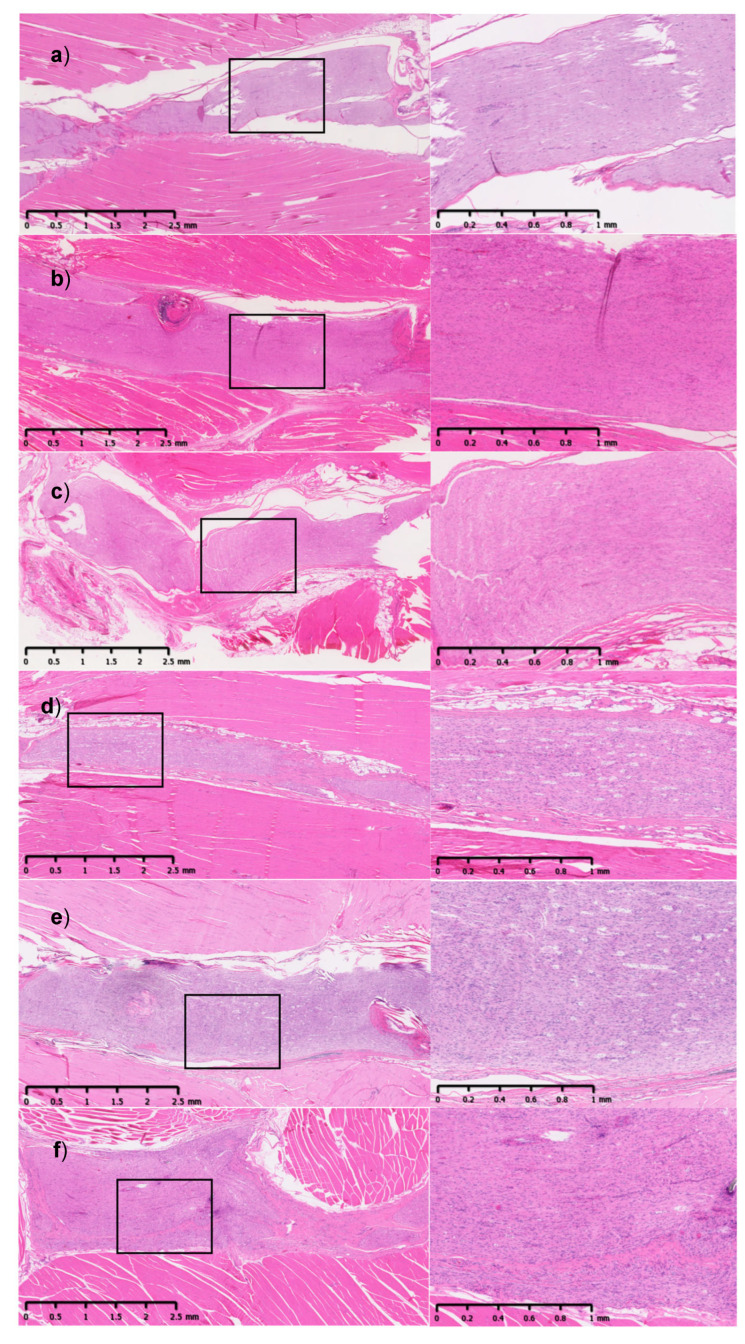
H&E-stained longitudinal sections of sciatic nerve and muscle (left 0.9x with a scale bar to 2.5 mm, right 2.5x with a scale bar to 1 mm) harvested at POD120. (**a**) UN: unoperated nerve, (**b**) AML: amnion monolayer, (**c**) ATL: amnion trilayer, (**d**) SIS: swine intestinal submucosa, (**e**) HAM: human amniotic membrane, (**f**) SR: suture repair. The black boxes represent the enlarged image areas that are shown on the right.

H&E-stained sections from the SR group and the membrane-repair groups showed an intense inflammatory reaction at POD30 in the tissues surrounding the neurorrhaphy site (Figure 3). Inflammation was more pronounced in the AML and HAM groups at this time point, with the membrane easily observed having an undulating shape around the nerve. The sections harvested at POD120 showed minimal presence of the membrane in the groups where these materials were used. For all groups, including suture repair, the inflammatory response was substantially decreased at 120 days (Figure 4).

**Figure 5:**
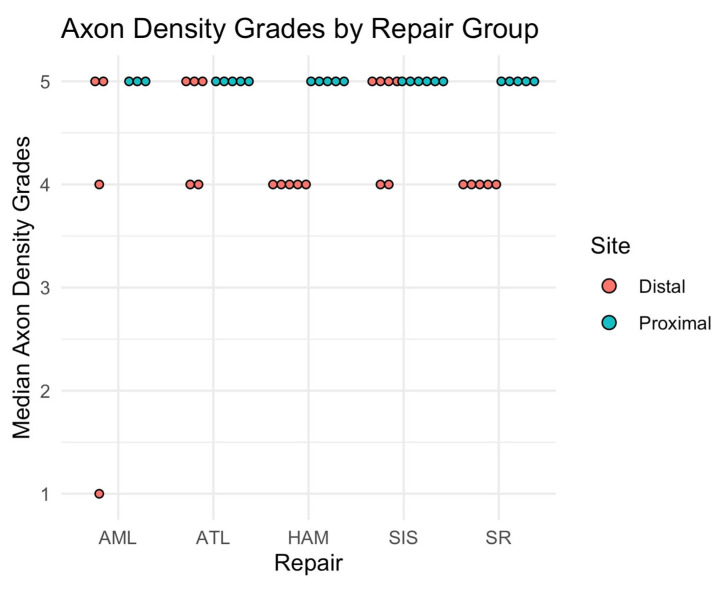
Dot plot showing the median axon density scores for each sample, by repair group, at proximal and distal locations at POD120. Note that samples were excluded from the groups due to poor quality of the sections.

**Figure 6:**
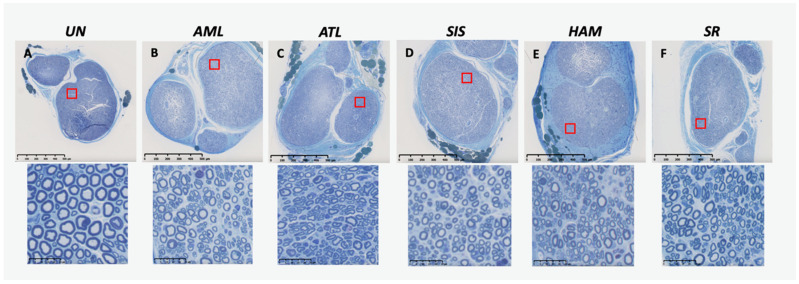
Toluidine blue-stained cross-sections of nerves from different groups. (**A**) UN: unoperated nerve, (**B**) AML: amnion monolayer, (**C**) ATL: amnion–chorion–amnion trilayer, (**D**) SIS: swine intestinal submucosa, (**E**) HAM: human amniotic membrane, (**F**) SR: suture repair. Magnification: 5x with a scale bar to 500 μm, and 80x with a scale bar to 25 μm. The red boxes represent the enlarged image areas that are shown below.

Histomorphometry of the nerves showed robust axon regeneration distal to the transection injury for all groups (Figure 5 and Figure 6).

No significant difference was found in median axon density scores between repair groups at the distal site (*p* = 0.10 for Kruskal–Wallis test). The median score at all proximal sites across all groups was five. Except for one sample repaired with ATL, the fascicle grades for all samples were one, meaning that ≥2/3 of the axons were contained within fascicles.

Differences in axon density scores between proximally and distally operated nerve sites within each group were also evaluated (Figure 5). In this model, proximal nerve scores serve as a measure of unaltered nerve health, while distal nerve sections serve as an assessment of nerve health following regeneration. The axon scores for distal nerve sections were significantly lower than scores for the proximal sections in the suture and human amnion repair groups (*p* < 0.05 for one-sided paired Wilcoxon test).

## 4. Discussion

The aim of this study was to compare the functional and histological outcomes of PTB nerve repairs using commercially available membranes to those using experimental PTB/HAM, as well as to standard microsurgical repairs with sutures. The goal was to assess whether the commercially available material is suitable for photosealing neurorrhaphy repairs, thereby facilitating its potential for clinical application.

In this rat sciatic model of nerve injury, repairs using PTB with commercially available collagen membranes had similar functional outcomes to repairs with PTB/HAM and SR, both at post-op day 30 and 120. Repairs with PTB trended towards higher SSI scores than those with suture, although this difference did not reach statistical significance. A mixed-effects model of SSI scores showed a significant positive effect from month of +3.23 units/month, but no significant effect from the repair group or an interaction between repair group and month. All groups trended towards improvement in SSI scores at similar rates.

Histological analysis of nerve sections at POD120 showed that the commercial membrane repairs trended towards superior axon regeneration compared to suture and human amnion repairs. Each of the commercial membrane repair groups had distal nerve samples that achieved axon density scores equal to the proximal control group; however, this was not the case in the suture and human amnion repair groups. Part of the benefit of PTB with membranes likely stems from its creation of a watertight seal around the nerve that is theorized to enhance regeneration by concentrating nerve growth factors and preventing axonal escape. Furthermore, PTB combined with a pliable and thin collagenous membrane does not seem to compress the nerve or hinder revascularization, as evidenced by comparable outcomes to SR. The trend for SSI scores to be better in PTB repairs than SR suggests non-inferiority of PTB and possible functional improvement.

A previous study of the biomechanical properties of PTB repairs with various commercial collagen membranes found that repairs with all tested membranes were able to withstand supraphysiological strain, with amnion-based membranes able to withstand slightly higher strain than SIS [22]. In this study, differences in membrane composition and resulting PTB bond strengths using various commercial collagen membranes and HAM did not translate into differences in functional recovery. PTB, therefore, appears to be a relatively robust method for providing adequate bond strength given sufficient collagen composition of the membrane.

In this study, a pronounced inflammatory response was observed across all groups at postoperative day 30, which appeared to diminish by day 120 upon qualitative microscopic examination. Based solely on a qualitative inspection of the histology, it appeared that HAM and AML exhibited greater inflammation than ATL, SIS, and SR at day 30. By POD120, both the inflammation and the PTB membranes, including the thicker ATL membrane, appeared to have largely disappeared and did not have any measurable effect on nerve regeneration or functional recovery. Although this qualitative inspection of inflammation in histologic slides might be considered a limitation, it is important to point out that this study builds on a larger set of previously reported positive results with PTB and serves as a screening of available commercial products to identify significant differences, utilizing qualitative data for this purpose.

The limitations of this study are the relatively small group size, which limited our ability to detect significant differences in outcomes between experimental groups, and the simplicity of the nerve transection model, which did not robustly challenge neural regeneration. Differences in nerve regeneration effects between various PTB membranes and suture repairs might be more apparent in a model involving a nerve gap injury. However, as a proof of principle for the use of commercially available collagenous membranes with PTB, the model proved adequate and provides foundational work for the evaluation of these membranes with PTB in a large nerve gap injury model in future studies.

Photochemical tissue bonding (PTB) with commercially available collagenous membranes has the potential to significantly impact clinical decision-making by providing a minimally invasive, effective alternative to traditional suturing for tissue repair. This approach leverages the regenerative properties of the enclosed environment provided by a sealed amniotic membrane or swine intestinal submucosa, which enhances the regenerative response by excluding scar tissue, preventing growing axons from escaping the repair site, and continuing growth factors elaborated within the repair site by Schwann cells and regenerating axons.

Numerous preclinical studies have demonstrated the benefits of PTB with human amnion for nerve repair. However, this study demonstrates that PTB can be successfully performed using various commercially available collagenous membranes, achieving comparable results to those of suture repair in a rat sciatic nerve transection injury model. The simplicity of the surgical technique achieved via photosealing decreases the complexity of neurorrhaphy compared to standard methods, potentially reducing operative time by requiring fewer or no microsutures for nerve repair and simultaneously enhancing the nerve regeneration process.

By virtue of its technical practicality and enhanced outcomes after nerve repair, it has broad applicability across various nerve repair scenarios, including direct nerve repairs of smaller digital nerves as well as larger nerves of the extremities, and in cases where grafting is required across nerve gaps, whether it be single-strand grafts or multiple cables. PTB is a versatile and promising technology that could transform surgical repair strategies for peripheral nerve injuries and enhance overall treatment outcomes. Clinical trials are needed to further characterize the regenerative effects of PTB in human subjects.

## 5. Conclusions

PTB with commercial membranes AML and SIS preserves nerve integrity and promotes functional recovery comparably to both suture repair and PTB with human amnion. This study highlights PTB as a robust method for nerve repair using commercially available collagenous membranes, which are better suited to clinical application than PTB with cryopreserved human amnion. These results strongly suggest that photosealing with these commercial membranes is at least as good as suture repair for peripheral nerve transection injuries and implementing PTB clinically has the potential to offer superior functional outcomes, as has been observed in prior animal work.

## Data Availability

The data presented in this study are available upon reasonable request from the corresponding author.
